# DISTINGUISHING SUBJECTIVE EXPERIENCE FROM OBJECTIVE FACTORS IN DECISION MAKING AND PERCEIVED EFFORT

**DOI:** 10.1093/geroni/igz038.2292

**Published:** 2019-11-08

**Authors:** Richard Gonzalez, Patricia Abbott, James Ashton-Miller, Jacqui Smith

**Affiliations:** University of Michigan, Ann Arbor, Michigan, United States

## Abstract

We use self-reported and behavioral data from the HomeLab to comment on the theoretical and methodological implications of integrating objective and subjective measures of experience. To illustrate, we will focus on two domains that vary in the nature of objective and subjective measurements examined. One domain will be decision making where subjective measures include subjective probability and utility and the respective objective measures include probability and actual outcomes. The second domain will be activities of daily living where the subjective measure is perceived effort and the objective measures include various data from sensor such as EDA (arousal) and EMG (muscle contraction). The presentation will discuss the benefits of conducting such research in a realistic standardized context such as the HomeLab, which is a fully connected, fully functioning apartment set up as a standardized lab in order to study activities of daily living.

